# A hybrid effectiveness-implementation study protocol to assess the effectiveness and chemoprevention efficacy of implementing seasonal malaria chemoprevention in five districts in Karamoja region, Uganda

**DOI:** 10.12688/gatesopenres.14287.2

**Published:** 2023-12-18

**Authors:** Richard Kajubi, Jennifer Ainsworth, Kevin Baker, Sol Richardson, Craig Bonnington, Christian Rassi, Jane Achan, Godfrey Magumba, Denis Rubahika, Jane Nabakooza, James Tibenderana, Anthony Nuwa, Jimmy Opigo

**Affiliations:** 1 Technical, Malaria Consortium, Kampala, Uganda; 2 Technical, Malaria Consortium, London, UK; 3 Department of Global Public Health, Karolinska Institute, Stockholm, Sweden; 4 National Malaria Control Division, Ministry of Health of Uganda, Kampala, Uganda

**Keywords:** Seasonal Malaria Chemoprevention, SMC, SPAQ, DP, chemoprevention efficacy, malaria, Karamoja, Uganda, East Africa, Southern Africa, cRCT, resistance markers

## Abstract

**Background:**

The World Health Organization (WHO) recommends seasonal malaria chemoprevention (SMC) with sulfadoxine-pyrimethamine and amodiaquine (SPAQ) for children aged 3 to 59 months, living in areas where malaria transmission is highly seasonal. However, due to widespread prevalence of resistance markers, SMC has not been implemented at scale in East and Southern Africa. An initial study in Uganda showed that SMC with SPAQ was feasible, acceptable, and protective against malaria in eligible children in Karamoja region. Nonetheless, exploration of alternative regimens is warranted since parasite resistance threats persist.

**Objective:**

The study aims to test the effectiveness of SMC with Dihydroartemisinin-piperaquine (DP) or SPAQ (DP-SMC & SPAQ-SMC), chemoprevention efficacy as well as the safety and tolerability of DP compared to that of SPAQ among 3-59 months old children in Karamoja region, an area of Uganda where malaria transmission is highly seasonal.

**Methods:**

A Type II hybrid effectiveness-implementation study design consisting of four components: 1) a cluster randomized controlled trial (cRCT) using passive surveillance to establish confirmed malaria cases in children using both SPAQ and DP; 2a) a prospective cohort study to determine the chemoprevention efficacy of SPAQ and DP (if SPAQ or DP clears sub-patent infection and provides 28 days of protection from new infection) and whether drug concentrations and/or resistance influence the ability to clear and prevent infection; 2b) a sub study examining pharmacokinetics of DP in children between 3 to <6 months; 3) a resistance markers study in children 3–59 months in the research districts plus the standard intervention districts to measure changes in resistance marker prevalence over time and finally; 4) a process evaluation.

**Discussion:**

This study evaluates the effects of SPAQ-SMC versus DP-SMC on clinical malaria in vulnerable children in the context of high parasite SP resistance, whilst informing on the best implementation strategies.

**Conclusion:**

This study will inform malaria policy in high-burden countries, specifically on utility of SMC outside the sahel, and contribute to progress in malaria control.

## Introduction

Seasonal malaria chemoprevention (SMC) is a very potent community-based intervention to avert malaria infections due to Plasmodium falciparum in areas such as the Sahel region, where malaria morbidity and mortality are high and malaria transmission is seasonal ^1– 3^. It entails the intermittent administration of antimalarials to at-risk populations during the period of greatest risk: usually coinciding with the rainy season. Since 2012, SMC has been recommended for children aged 3-59 months using sulfadoxine-pyrimethamine (SP) and amodiaquine by the World Health Organization (WHO) ^4^. Owing to concerns over widespread parasite resistance to SP and AQ across east and southern Africa, the Sahel region of west and central Africa has historically been prioritised for the scale-up of SMC. In 2020, SMC was implemented in 13 Sahelian countries targeting over 33 million children ^5^. Consolidated guidelines for malaria published by WHO in 2023 no longer provide specific parameters for the deployment of SMC in terms of seasonality, drug regimen or therapeutic efficacy of the antimalarials used, emphasising the importance of context and local evidence ^6^.

There is therefore a need to explore if SMC can be a viable malaria prevention strategy in new geographies, including those where resistance to SP and AQ is high, especially since the definition is not clear regarding the relation between resistance and the effectiveness of SMC. Studies have shown that across east and southern Africa P falciparum responses to SP are seriously compromised ^7– 9^, and SP is no longer recommended for treatment of malaria episodes. Several mutant alleles that confer parasite resistance to SP are commonly found in the region, including in P falciparum dihydrofolate reductase (pfdhfr) gene at codons 51, 59 and 108, and in P falciparum dihydropteroate synthase (pfdhps) gene at codons 437 and 540 ^10^. The combination of three mutations in pfdhfr (51I, 59R, and 108N) and two in pfdhps (437G and 540E) leads to an intermediate level of SP resistance ^11^. Addition of either pfdhfr164L or pfdhps 581G leads to higher-level SP resistance ^11^. These additional mutations have been rare in Africa, but some reports have noted moderate prevalence of the pfdhfr 164L mutation in parasites from south-western Uganda ^12– 15^, and of the pfdhps581G mutation in parasites from Uganda and Tanzania ^8, 9, 16^. For another malaria chemoprevention strategy, intermittent preventive treatment in pregnancy (IPTp), which also uses SP, it has been suggested that parasite resistance may undermine the effectiveness of the intervention ^17^. Markers associated with amodiaquine resistance have also been found, including in the P falciparum multidrug resistance 1 (Pfmdr1) gene and the chloroquine resistance transporter (PfCRT). Even if the effectiveness of SMC in the context of high parasite existence can be demonstrated, it will also be important to identify alternative SMC drug regimens that could replace SPAQ in the future.

The Uganda National Malaria Reduction Strategic Plan 2021-2025 recommends the implementation of SMC in areas with highly seasonal malaria to accelerate progress towards malaria elimination ^18^. In response to this recommendation, the National Malaria Control Division (NMCD) and Malaria Consortium are conducting SMC implementation research in Karamoja subregion. In 2021, SMC using SPAQ was implemented in two districts with a combined target population of 85,000 children 3–59 months. Five monthly SMC cycles were implemented between May and September. Village Health Teams, a recognised cadre of community health workers, served as community distributors, with supervision provided by health facility-based health workers. The intervention was found to be feasible and acceptable, with high coverage achieved through the standard door-to-door delivery mechanism. There were also indications that the intervention was effective in preventing malaria cases among targeted children. However, there remains a need to generate robust evidence of effectiveness using appropriate study designs. There is also a need to further explore if SMC using SPAQ can provide a sustained, prolonged protective effect against malaria in the context of parasite resistance.

In 2022, SMC will be implemented in eight districts of Karamoja, targeting 270,000 children 3–59 months. As in the previous year, five monthly SMC cycles will be implemented using a door-to-door delivery model. The majority of children will receive SMC with SPAQ, while around 15,000 children will receive SMC with dihydroartemisinin-piperaquine (DP). As there is currently no age-based dosing regimen for SMC, DP will be given according to a weight-based dosing regimen. In Uganda, DP is used as second-line treatment for uncomplicated malaria and remains highly effective and safe for the treatment of uncomplicated malaria ^19, 20^. It has also been used successfully in mass drug administration and to reduce malaria incidence in emergency settings. DP could be an alternative SMC drug regimen primarily due to its long half-life. However, there is insufficient evidence of its tolerability in very young children as safety studies have typically involved only children above 6 months.

## Protocol

### Aims and objectives

This study phase aims to test the feasibility, effectiveness, and chemoprevention efficacy of SMC with SPAQ and DP in Karamoja region in Uganda, where malaria transmission is highly seasonal, and inform malaria policy in Uganda. Specific objectives include: 1) to determine the effectiveness of SMC with SPAQ and DP in terms of its reduction in incidence of malaria infection among children aged 3–59 month for SPAQ and children 6–59 months for DP; 2a) to conduct a prospective chemoprevention efficacy cohort study to determine if SPAQ and DP clear existing parasitemia and provide 28 days of protection from infection and whether drug concentrations and/or SPAQ or DP drug resistance influence the protection duration of these two SMC regimen; 2b) to assess the safety of DP in children 3–6 months through pharmacometric analysis; 3) to conduct a resistance markers study in children 3–59 months in the two research districts plus the three standard intervention districts to measure changes in resistance marker prevalence over time; and 4) to assess the impact of the SMC implementation model, determining process, costing and acceptability outcomes for the intervention.

## Methods

### Study design

This type 2 hybrid effectiveness-implementation study uses a convergent mixed-methods approach, as previously described in other similar studies conducted by our group ^21, 22^. SMC with SPAQ and DP will be implemented in five monthly cycles between June 2022 and October 2022 in five districts of Karamoja. Over 85,795 children aged 3–59 months, in Kotido and Moroto for phase 1 of the study, will continue to receive SMC in phase 2, and in addition, 60,000 children in three districts (Nabilatuk, Amudat, and Nakapiripirit) will receive SMC for the first time. The research will involve the following components: 1) Conducting a cluster randomized controlled trial (cRCT) using passive surveillance to establish confirmed malaria cases among participating children; 2) conducting a prospective chemoprevention efficacy cohort study to determine whether SPAQ and/or DP provides 28 days of protection from infection, and whether drug concentrations and/or resistance influence the duration of protection; 3) conducting a resistance markers study in children aged 3–59 months in the three research districts plus the two standard intervention districts to describe changes in resistance marker prevalence over time; and 4) conduct a process evaluation of the SMC implementation looking at cost and process outcomes ^6^.

### Study setting

**Figure 1.**
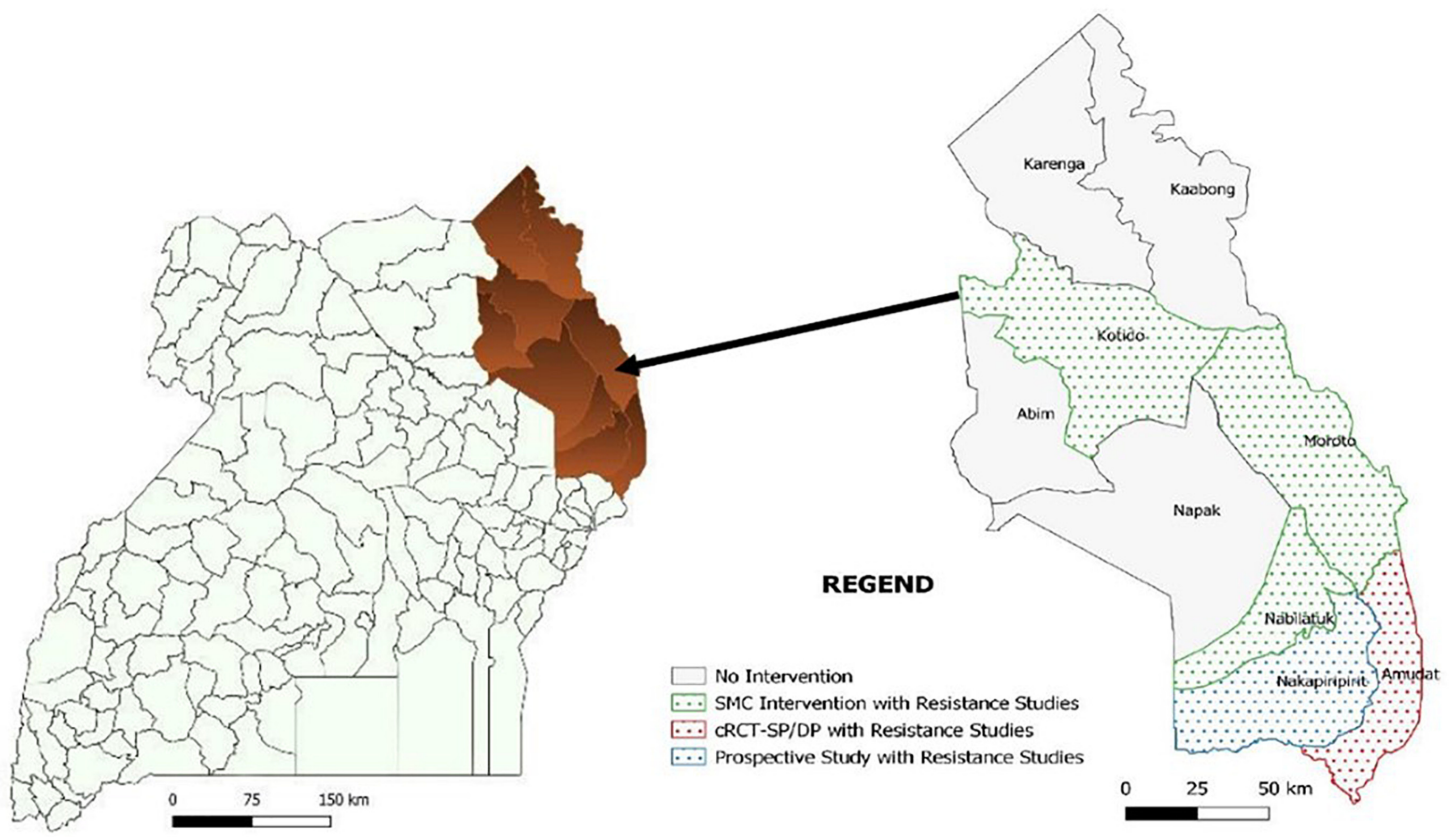
Area of implementation of Year 2 SMC in Uganda.

The study will be conducted in five districts of Karamoja region as shown in Figure 1 below. The cRCT will be conducted in Amudat. The chemoprevention component will be conducted in Nakapiripirit because SMC has not been previously implemented in this location therefore drug resistance selection pressure from a previous year of SMC implementation will be avoided or minimized and malaria rates are expected to be higher allowing for a greater detection of breakthrough infections. The resistance markers component will be conducted in all five districts inclusive of Nabilatuk, Kotido and Moroto, as well as the process evaluation for assessment of the SMC implementation model.

### Population

Eligible participants for receiving SMC will be male or female children with no fever, aged 3–59 months for any of the SMC cycles, staying in the districts of Kotido, Moroto and Nabilatuk districts (where delivery is continuing from Phase 1 and where coverage surveys will be conducted in Phase 2), and randomly-selected areas in Amudat and Nakapiripirit.

Additionally, other key stakeholders who will be sampled include health workers participating in implementing SMC, health officials at different levels of the health system, community leaders, and caregivers of children below ten years of age, (see Table 1 for estimated population sizes).

**Table 1.** Sample sizes for each objective.

Objective	Study population	Estimated sample size
1	cRCT: Sample of eligible children in Amudat district of age 3–59 months, resident in the study area for at least a month, planned for DP and SPAQ	4,270
2a	Chemoprevention efficacy cohort study – children under 3–59 months for SPAQ and 6-59 months for DP	1,250
2b	Safety of DP in 3–6 month old children through pharmacometrics to low dose DP.	30
3	Resistance Marker study – children under 5	1,500
4	Assessment of the SMC implementation model, deciding the process, costing and acceptability outcomes of the intervention	144
**Total number of participants:**		**7,194**

### Recruitment and data collection

***cRCT component.*** In the control arm, one eligible child will be recruited at random in each household of which, researchers from selected communities will have randomly selected. Informed consent will be obtained from caregivers, then children will be enrolled and their eligibility confirmed using a short baseline questionnaire to collect individual data on each child.

For the intervention arms, VHTs will be followed by a researcher as they administer SMC. Households in selected communities will be randomly sampled from those visited by the VHTs. Before SMC is initiated, one eligible child will be recruited, and a short baseline questionnaire will be administered to collect individual data and to confirm their eligibility. Informed consent will be obtained from caregivers and then a questionnaire used for collecting individual child data, to which all household questions from the end-of-round survey will be added (e.g. on household wealth, language, migration status, education and level of literacy of caregivers and heads-of-household, etc.), and to determine their eligibility. Blood samples will be taken for haemoglobin concentration measurement using a Hemocue machine.

The primary endpoint will be recorded with passive surveillance conducted by study clinicians in intervention and control areas throughout the five-month study period.

Children recruited into the study presenting at clinics or to VHTs will be identified using information on their SMC record cards, and data on clinic visits including suspected malaria cases and results of RDTs will be matched to baseline questionnaire data to build a database for analysis. Date of clinic attendance and confirmation of malaria cases using rapid diagnostic tests will be recorded to allow for calculation of follow-up time and time to malaria events for fitting of Cox proportional hazards regression model. This will be facilitated through matching of codes on children's SMC cards with records of the same codes in clinic logbooks.

**Figure 2.**
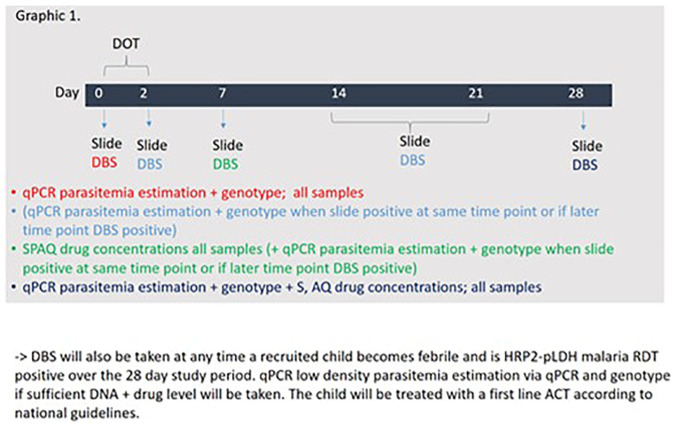
Planned sample collection time points and subsequent sample processing sequence.

***Chemoprevention efficacy cohort study.*** Children will be screened at the community level by research assistants with the help of VHTs and referred to a designated study clinic for further assessment and non-randomized recruitment. At the study clinic, study clinicians will confirm eligibility criteria through conversation with the caretaker and obtain informed consent. Enrolled children will receive SMC via directly observed therapy (DOT) over the first three days and will have malaria thick smears and dry blood spots (DBS) taken at day 0, 2, 7, 14, 21 and 28 throughout and after administering a single cycle of SPAQ or DP. In addition, the study clinician will assess if the child received other treatment or experienced any problem during all the scheduled sample collection days (Day 0, 7, 14, 21, 28) as well as bed net usage. Caregivers and guardians will also be reminded at each visit to seek care at a health facility or from a community health worker if the child becomes febrile. Children who become febrile and are confirmed on HPR2-pLDH RDT between day 0 and day 28 will have an additional DBS taken and then treated using standard guidelines. All DBS samples will be processed for all of the SMC drugs on days 7 and for sulfadoxine, amodiaquine and piperaquine for day 28. Individual surveys on day 28 after the final DBS is taken will be conducted to determine if the child received other treatment or experienced illness over the past month of study implementation. On day 0 and day 28, they will also be processed to ascertain low density parasitaemia estimates through quantitative polymerase chain reaction (qPCR). Figure 2 below depicts the planned sample collection time points and subsequent sample processing sequence). We aim to recruit participants from Nakapiripit, one of the two research districts.

***Resistance markers study.*** We will monitor changes of SP, AQ, resistance in the three intervention districts, receiving SMC using only SPAQ (Nabilatuk, Kotido and Moroto) plus the two research districts where the SMC will be implemented using both SPAQ and DP (Amudat and Nakapiripirit), while the DP resistance will be monitored only in the later. A baseline cross-sectional survey will be conducted based at the health facilities before SMC project implementation as well as an endline survey following one complete round of SMC distribution to measure the prevalence of molecular markers associated with resistance to SP/DP in symptomatic children under five years of age with a positive RDT attending selected health facilities in the intervention and control areas.

The key markers of resistance to be monitored are: dihydrofolate reductase (dhfr): codons 108, 51, 59 and 164; dihydropteorate synthetase (dhps): codons 431, 437, 540, 581 and 613; Pf chloroquine resistance transporter gene (pfcrt): codons 72-76; Pf multidrug resistance gene 1 (pfmdr1): codons 86, 184 and 1246, Plasmepsin 2 (plm2) and K13 mutations of significance

***Process evaluation component.*** Once interview participants are identified for potential recruitment, they will be approached by a research assistant who will share more information about the study and invite their participation. If willing, a meeting will then be set up at a time and location that is convenient for the individual, where further information will be shared about the study including a written information sheet provided to all participants.

In-depth interviews and focus group discussions based on topic guides will be utilized to explore insights on SMC including acceptability and thoughts regarding the experience of implementation.

### Data analysis

***cRCT component.*** We will accomplish data analysis using Stata and excel. The proportion command, with 95% confidence intervals calculated using a logit transform will be used to compute coverage. Population size weights will be applied with the svy: command as appropriate for the estimates of coverage indicators whereby a self-weighting sample was not possible to achieve. The pertinent indicators will be calculated using proportions per district and an average across both districts. The confidence interval (CI) of 95% will be used to provide a range of values around the estimate within which selected result will be expected to fall.

Data from HMIS will be retrospectively analysed to identify epidemiological trends in malaria in the study districts by age group. Summary statistics will be computed and data will be graphically presented by month over the study period.

***Chemoprevention efficacy cohort study.*** Relevant mutations distributions and proportions will be analysed comparing parasitological efficacy between groups of mutations. Analysis of the blood drug concentrations will be done as a cohort by mean, median and standard deviation, and statistical tests of their associations with treatment outcomes, precisely drug levels on day seven. Outliers with low levels of drug concentration based on the outcome measures described will be focused upon. Positivity on day 28 will be associated with the antimalarial drug resistance genotype. Chemoprevention failure rates will be reported on as the cumulative failure rate using Kaplan-Meier analysis of the proportion. Time to event analysis will be incorporated to assess duration of SMC protection.

***Resistance markers study.*** The prevalence of Pfdhps, Pfdhfr Pfcrt and Pfmdr1 and K13 alleles will be measured in parasites obtained from smear positive children. Additionally, the copy number of Pfplm2 will also be assessed.

***Process evaluation component.*** The data will be analysed thematically. Both key informant interviews and FGDs will be audio-recorded and thereafter transcribed verbatim i.e. in the real words (or equivalent translations) used by the informants. These will be organised according to findings relating to SMC acceptability, implementation, and views and experiences with SMC more broadly. Initially, open, descriptive coding will be used in order to ensure interpretations are grounded in participants’ accounts as much as possible, and that findings are generated inductively, as well as exploring pre-determined areas of investigation (for example, relating to acceptability of SMC). The transcribed data will be entered into Nvivo 12 for analysis and coded based on both pre-determined themes as well as those that emerged from the data. Codes and themes will continuously be refined as the analysis progresses. The coded text under each code / theme will provide the basis for determining the patterns and meanings in the data, comparing across data from different respondent groups, and for making arguments and conclusions. Relevant quotations will also be identified and included in the analysis report.

### Data management

For the cRCT component (1), using computer aided personal interview (CAPI), which allows for in-field data entry and server synchronisation, data collected will be verified for quality assurance purposes by the quality assurance officer in field and uploaded daily to the SurveyCTO platform. The uploaded files will undergo additional consistency checks, cleaned and saved into Stata. Extensive data cleaning will be done at the end of the fieldwork.

For the chemoprevention efficacy and safety studies, all clinical data will be recorded onto standardized case record forms (CRFs) by IDRC study clinicians. Data will then be entered directly from CRFs into a computerized database or transferred from the CRFs onto standardized data extraction forms and then into a computerized database. All computerized data will be double entered to verify accuracy of entry.

For the resistance markers study, data will initially be managed at health facilities-level by field workers with support from the IDRC Data Management Team (DMT). They will send the pVHTr questionnaires to the IDRC once sample collection has been completed. Data will then be entered by a data entry clerk using specific software for clinical data management. Rigorous consistency checks will be created to reduce errors during data entry.

For the process evaluation, data will be stored without any participant-identifying information (with use of pseudonyms and removal of job title or other identifying information), in a password-protected format. Raw data collected and analysed will not be shared with anyone other than the researchers of this study.

### Eligibility criteria

Inclusion and exclusion criteria for each objective are detailed in Table 2 below.

**Table 2.** Eligibility Criteria.

Inclusion criterion	(1)	(2)	(3)	(4)
Children between 3–59 months	x	x	x	
Being resident in the study area	x	x	x	
Afebrile with no other malaria associated symptoms in the past 48 hours or at time of recruitment	X	X		
Consent to participate in the study obtained	X	X	X	
Willingness and ability of the child’s guardians to comply with the study protocol for the duration of the study including attending a designated health centre if their child has malaria symptoms during the data collection period	x	X		
Can comply with 3 days DOT of standard SPAQ or DP regimen (day 0–2		x		
Clinical signs and symptoms suggestive of malaria infection: fever (axillary temperature ≥37.5ºC) or history of fever in the preceding 24 hours.			X	
Positive RDT			x	
A person aged 18 years or older with primary or caring responsibility for the feeding and daily care of at least one child aged 3–59 months, who has been resident in Nabilatuk, Kotido, Moroto, Amudat or Nakapiripirit prior to the beginning of the SMC pilot				x
Community members, community leaders, religious leaders				X
Village Health Teams (VHTs) involved in the distribution of SMC in Nabitatuk, Kotido, Moroto, Amudat and Nakapiripirit				X
Community Health Workers, Community Distributors				X
District level health officials (District Health Officers, Malaria Focal Persons)				X
Provincial level health officials				X
Implementing partners				X
National level policy makers				X
Gender experts and/or gender advocates				x
Exclusion criterion	(1)	(2)	(3)	(4)
Malaria symptoms (tympanic fever ≥ 37.5°C or history of fever in past 48 hours)	X	X		
Known allergy to medicine provided	X	X		
Receiving a sulfa-based medication for treatment or prophylaxis, including co-trimoxazole (trimethoprim– sulfamethoxazole)	X	X		
Individuals receiving azithromycin due to the antimalarial activity of azithromycin	X	x		
Severe malnutrition according to WHO guidelines	X	X		
Recruited in any other SMC study element	X	X		
Children with HIV or ARV use	X	X		
Children with a history of severe adverse reaction to SP, AQ or DP or have used SP within the previous month before recruitment	x	X		
Treatment of uncomplicated malaria with DP in the past 28 days		X		
Chronic illness of any kind		X		
Recruited in cRCT or any other studies		X		
Treatment with an ACT in previous 2 weeks		X		
Any signs or symptoms of severe malaria			X	
Negative RDT			x	
Any individual who refuses consent to participate	x	x	x	X
Individuals aged under 18 years				x

### Ethical considerations

Ethical approval for this study was obtained from the Mbale Regional Referral Hospital-REC on 28 April 2022 (Ref: MRRH-2022-168) and by the Uganda National Council for Science and Technology (UNCST) on 5 May 2022 (Ref: HS2212ES). Only participants that meet the inclusion criteria and whose caregivers provide written informed consent will be included in this study. The trial has been registered on clinicaltrials.gov (Ref: NCT05323721).

### Study status

Research activities commenced in June 2022 after approval of Ethics Committees. Data collection has been completed and cleaning and preliminary analysis is on-going. Final analysis is expected to be completed by April 2023.

### Dissemination

Malaria Consortium will develop a research uptake plan to help the study team identify relevant stakeholders and consider appropriate messages and activities, along with respective budgets and timelines, to ensure these stakeholders are kept engaged and informed of progress throughout the research cycle. Results will be disseminated through agreed peer-review publications and international conferences. There will also be collaboration with the Ministry of Health National Malaria Control Division to disseminate findings nationally.

## Conclusions

This study will contribute strong evidence on the effectiveness of SMC in the context of high parasite resistance in areas outside the Sahel which had previously not been prioritized for scale-up, using appropriate study designs. The findings will be crucial for future SMC research and deployment by understanding if drug dosing or drug choice may need to be altered to provide continued efficacious SMC in the local context. Ultimately, this will influence policy change in malaria control.

## Data Availability

No data are associated with this article.
