# Supplementary material for: A hybrid effectiveness-implementation study protocol to assess the effectiveness and chemoprevention efficacy of implementing seasonal malaria chemoprevention in five districts in Karamoja region, Uganda
Source: Gates Open Res. 2023 Dec 18;7:14. Originally published 2023 Jan 30. [Version 2] doi: 10.12688/gatesopenres.14287.2 (PMC10774186; doi:10.12688/gatesopenres.14287.2)
Supplement: Peer review history

# Reviewer report: Reviewer response for version 2

**DOI:** 10.21956/gatesopenres.16472.r35688

**Published:** 2024-02-26

**Authors:** Robertus Dole Guntur, Ph.D

**Affiliations:** 1 Department of Mathematics Faculty of Science and Engineering, Nusa Cendana University, Kupang, East Nusa Tenggara, Indonesia

Question 1

 If you could provide the flow chart for recruiting your participants, it would be great to improve the article. The flow chart is a kind of picture so it could not add the number of words to this article. Then, the authors said, “we make the flow chart available in a future publication after data collection”.  Meanwhile on the study status section of this protocol paper, the author said that “Data collection has been completed “. Would you please to clarify this?

 Question 2

 The rationale in determining sample size is very important in a study. The data in Table 1 is like a magic number if the authors could not provide the reasons in choosing those numbers.

 Question 3

 The questionnaire is an important aspect in assessing this article as the authors collected individual data from the children. The consistency between your statement in the article and the questionnaire is important to be evaluated!!! The authors could include the questionnaires as the supplementary material for this submission.

 Question 4

 Plan analysis is one of the essential components on a protocol paper. The authors could provide general plan on data analysis to achieve your aims as each aim of this study has a different statistical method.

Is the study design appropriate for the research question?

Yes

Is the rationale for, and objectives of, the study clearly described?

Yes

Are sufficient details of the methods provided to allow replication by others?

No

Are the datasets clearly presented in a useable and accessible format?

Not applicable

Reviewer Expertise:

Epidemiology and Biostatistics, Malaria

I confirm that I have read this submission and believe that I have an appropriate level of expertise to confirm that it is of an acceptable scientific standard, however I have significant reservations, as outlined above.

# Reviewer report: Reviewer response for version 2

**DOI:** 10.21956/gatesopenres.16472.r35687

**Published:** 2024-01-31

**Authors:** Fatima Nogueira

**Affiliations:** 1 Universidade Nova de Lisboa, Lisbon, Lisbon, Portugal

Changes were made.

Is the study design appropriate for the research question?

Partly

Is the rationale for, and objectives of, the study clearly described?

Yes

Are sufficient details of the methods provided to allow replication by others?

No

Are the datasets clearly presented in a useable and accessible format?

Not applicable

Reviewer Expertise:

malaria drug resistance

I confirm that I have read this submission and believe that I have an appropriate level of expertise to confirm that it is of an acceptable scientific standard.

# Reviewer report: Reviewer response for version 2

**DOI:** 10.21956/gatesopenres.16472.r35689

**Published:** 2024-01-31

**Authors:** Abena Poku-Awuku

**Affiliations:** 1 Medicines for Malaria Venture, Geneva, Geneva, Switzerland

Keywords: I'm not sure if it makes sense to state both Seasonal Malaria Chemoprevention and SMC as two separate keywords.

 Conclusion in abstract: Sahel should start with a capital S

 Introduction: *Plasmodium falciparum* and all other mentions of *P falciparum *or scientific names must be italicized.

 Line 7 of introduction: West and Central Africa should start with capital letters as they are the names of the regions; western and southern Africa, not as they are not names

 Recruitment and data collection (resistance markers)- later in line 3 of this section should be "latter"

 Process evaluation component: Please state out what FGD is before using the acronym

 Overall, the script is fine but requires proof reading.

Is the study design appropriate for the research question?

Yes

Is the rationale for, and objectives of, the study clearly described?

Yes

Are sufficient details of the methods provided to allow replication by others?

Yes

Are the datasets clearly presented in a useable and accessible format?

No

Reviewer Expertise:

Malaria, advocacy

I confirm that I have read this submission and believe that I have an appropriate level of expertise to confirm that it is of an acceptable scientific standard.

# Reviewer report: Reviewer response for version 1

**DOI:** 10.21956/gatesopenres.15598.r33878

**Published:** 2023-07-17

**Authors:** Robertus Dole Guntur, Ph.D

**Affiliations:** 1 Department of Mathematics Faculty of Science and Engineering, Nusa Cendana University, Kupang, East Nusa Tenggara, Indonesia

The method section lacks details. The author should provide as many details as possible in the methods section.

1. Please provide a flow chart of a recruitment strategy for your participants.
2. Please provide sample size calculation to justify your sample size in Table 1.
3. The authors said that the questionnaire is used for collecting individual child data. Where is your questionnaire? Please attach as supporting documents.
4. In the analysis section, the procedure on how the authors analyse the data of 7,194 participants is not clear. How do you analyse your data to achieve your aim of study? Please breakdown your analysis plan following your objective 1 to 4. How do you analyse the socio-demographic data of your participants? Furthermore, in the part of resistance markers study, what is your statistical analysis to achieve the objective number 3?

Is the study design appropriate for the research question?

Yes

Is the rationale for, and objectives of, the study clearly described?

Yes

Are sufficient details of the methods provided to allow replication by others?

No

Are the datasets clearly presented in a useable and accessible format?

Not applicable

Reviewer Expertise:

Epidemiology and Biostatistics, Malaria

I confirm that I have read this submission and believe that I have an appropriate level of expertise to confirm that it is of an acceptable scientific standard, however I have significant reservations, as outlined above.

# Response

**Published:** 2023-07-25

**Authors:** Richard Kajubi

**Affiliations:** Malaria Consortium, Uganda

**Dear Robertus Dole Guntur,**

**Thank you for your interest and valuable feedback to our article. Please see below responses to your comments.**

 The method section lacks details. The author should provide as many details as possible in the methods section.

1. Please provide a flow chart of a recruitment strategy for your participants.
**Thank you for your comment. We appreciate your interest in having a flow chart of our recruitment strategy. However, due to space limitations in th** **is protocol paper, we were unable to include visual content like flow charts. We understand the value of a recruitment flow chart for readers to better understand the study's participant selection process.** **However, we provid** **ed a detailed description of the recruitment strategy in the "Methods" section to enhance transparency and clarity. Your suggestion is valuable, and we make the flow chart available** **in a future publication after data collection. **
2. Please provide sample size calculation to justify your sample size in Table 1.
**Thank you for your valuable feedback. We agree that providing a sample size calculation is essential to justify the chosen sample size in Table 1. However, the sample size was determined based on prior research in the field, feasibility constraints, and the statistical requirements for detecting meaningful effects in our study. Due to word count limitations in the article, we were unable to include the sample size calculation in the main text.** **To address this concern, we will include a detailed sample size calculation in the supplementary materials or as an online appendix to ensure transparency and support the robustness of our study. This will allow readers to better understand the rationale behind the selected sample size and the statistical power of our analysis.**
3. The authors said that the questionnaire is used for collecting individual child data. Where is your questionnaire? Please attach as supporting documents **Thank you for your comment and interest in our study. We apologize for any confusion. While we understand the importance of providing the questionnaire for transparency and reproducibility, due to the word limit constraints, we were unable to include the complete questionnaire in the article.** **However, we want to assure you that the questionnaire used in the study is available for reference and upon request. To ensure accessibility and facilitate reproducibility, we will make the questionnaire available as supplementary material or on request by contacting the corresponding author.**
4. In the analysis section, the procedure on how the authors analyse the data of 7,194 participants is not clear. How do you analyse your data to achieve your aim of study? Please breakdown your analysis plan following your objective 1 to 4. How do you analyse the socio-demographic data of your participants? Furthermore, in the part of resistance markers study, what is your statistical analysis to achieve the objective number 3? **Thank you for your valuable feedback and for seeking clarification on our data analysis procedures. We understand the importance of providing a clear and detailed analysis plan to support the study's objectives.** **However, due to the word limit constraints, we were unable to include** **that information in th** **is article** **. We will ensure that these details are available in all subsequent publications with regard to each of the objectives.**

# Reviewer report: Reviewer response for version 1

**DOI:** 10.21956/gatesopenres.15598.r33622

**Published:** 2023-06-28

**Authors:** Fatima Nogueira

**Affiliations:** 1 Universidade Nova de Lisboa, Lisbon, Lisbon, Portugal

1. Include the meaning of abbreviations in full at the beginning of the document, such as SPAQ.
2. Homogenize the reference to the therapeutic combinations used.
3. In the “Methods Study design” refer to and comply with the WHO guidelines (WHO-UCN-GMP-2023.01-eng).
4. Consider including gametocyte count/carriage.

Is the study design appropriate for the research question?

Partly

Is the rationale for, and objectives of, the study clearly described?

Yes

Are sufficient details of the methods provided to allow replication by others?

No

Are the datasets clearly presented in a useable and accessible format?

Not applicable

Reviewer Expertise:

malaria drug resistance

I confirm that I have read this submission and believe that I have an appropriate level of expertise to confirm that it is of an acceptable scientific standard, however I have significant reservations, as outlined above.

# Response

**Published:** 2023-07-25

**Authors:** Richard Kajubi

**Affiliations:** Malaria Consortium, Uganda

**Dear Fatima Nogueira,**

**Thank you for your valuable insights and feedback. Please see the responses to your concerns below.**

 Include the meaning of abbreviations in full at the beginning of the document, such as SPAQ.

**Thank you for your suggestion. We recognize the importance of providing clarity to readers by including the full meaning of abbreviations at the beginning of the document. Please note that We used the wording "sulfadoxine-pyrimethamine and amodiaquine" at the beginning of both the abstract and the main body of the article.**

 Homogenize the reference to the therapeutic combinations used.

**Thank you for your suggestion to homogenize the reference to the therapeutic combinations used. We understand the importance of maintaining consistency and clarity in scientific writing. We will review all references to therapeutic combinations throughout the article and ensure that they are presented in a uniform manner. This may involve standardizing the naming conventions, abbreviations, or any other relevant details related to the therapeutic combinations.**

 In the “Methods Study design” refer to and comply with the WHO guidelines (WHO-UCN-GMP-2023.01-eng).

**Thank you for your suggestion to refer to and comply with the WHO guidelines (WHO-UCN-GMP-2023.01-eng) in the "Methods Study design" section. We recognize the significance of adhering to established guidelines to ensure the credibility and validity of our research.We will review and add the specific reference to the WHO guidelines (WHO-UCN-GMP-2023.01-eng) and describe how our study design aligns with the recommended protocols and methodologies outlined in the guidelines.**

 Consider including gametocyte count/carriage.

**Thank you for your suggestion to include gametocyte count/carriage in the study. We recognize the importance of assessing gametocyte count/carriage as a significant parameter in malaria research, especially in understanding transmission dynamics. Unfortunately, in this particular study, we did not include gametocyte count/carriage as one of the study variables hence we did not collect that data.**

# Reviewer report: Reviewer response for version 1

**DOI:** 10.21956/gatesopenres.15598.r33624

**Published:** 2023-06-15

**Authors:** Abena Poku-Awuku

**Affiliations:** 1 Medicines for Malaria Venture, Geneva, Geneva, Switzerland

The discussion and conclusion put in the abstract are too vague and do not reflect the outcomes found in the body of the article.

 Introduction:

 Lines 1-3: It would be good to state upfront that SMC is widely implemented in the Sahel and has been used only there. Lines 1-3 make it seem as if SMC can be implemented in all malaria-endemic areas.

 Lines 8-9: WHO's implementation manual published in 2023 provides parameters for seasonality. Please check and refer to accordingly.

 Lines 13-15: I would remove the part of the sentence that says "the definition is not clear regarding relation between resistance and effectiveness of SMC" because it contradicts what is said 2 sentences later. 

 Recruitment and data collection: Line 6: is it only one child or one child per household?

 Line 10: what were the end of round demographic survey results used for?

 Process evaluation component: Line 5 typo: possible60; line 9: "progresses" not "progressed".

 This paper would be stronger and more complete if the final data results would be available. If this is not ready, I'm not clear if the objective of the study has been achieved or if results to substantiate the objectives can be proven.

Is the study design appropriate for the research question?

Yes

Is the rationale for, and objectives of, the study clearly described?

Yes

Are sufficient details of the methods provided to allow replication by others?

Yes

Are the datasets clearly presented in a useable and accessible format?

No

Reviewer Expertise:

Malaria, advocacy

I confirm that I have read this submission and believe that I have an appropriate level of expertise to confirm that it is of an acceptable scientific standard, however I have significant reservations, as outlined above.

# Response

**Published:** 2023-07-25

**Authors:** Richard Kajubi

**Affiliations:** Malaria Consortium, Uganda

**Dear Abena Poku-Awuku,**

**Thank you for your valuable feedback. Please see the responses to your concerns below.**

 The discussion and conclusion put in the abstract are too vague and do not reflect the outcomes found in the body of the article.

**Thank you for your valuable feedback. We appreciate your concern regarding the clarity of the discussion and conclusion in the abstract. While we understand your point, the purpose of the abstract is to provide a concise overview of the study and its key findings. However, this being a protocol article, we had not yet collected complete data at the time of submission hence we didn’t have any key findings to discuss and make a more compelling conclusion. We will ensure to do that in the subsequent manuscript after all data collection is complete.**

 Introduction:

 Lines 1-3: It would be good to state upfront that SMC is widely implemented in the Sahel and has been used only there. Lines 1-3 make it seem as if SMC can be implemented in all malaria-endemic areas.

**Thank you for your insightful comment. We appreciate your suggestion to clarify the geographic scope of SMC implementation in the article. However, we did clarify that SMC applies to all malaria-endemic areas where transmission is seasonal, and the same paragraph clearly stipulates that SMC was implemented in 13 Sahelian countries and had been used only there until we conceived these studies. We feel that can suffice.**

 Lines 8-9: WHO's implementation manual published in 2023 provides parameters for seasonality. Please check and refer to accordingly.

**Thank you for bringing this to our attention. We appreciate your diligence in pointing out the availability of the WHO's implementation manual published in 2023, which provides parameters for seasonality in the context of our study. However, at the time of submitting our protocol, some of the study justifications had been based on Consolidated guidelines for malaria published by WHO in 2022, hence we felt that it was more appropriate to cite the 2022 guidelines.**

 Lines 13-15: I would remove the part of the sentence that says "the definition is not clear regarding relation between resistance and effectiveness of SMC" because it contradicts what is said 2 sentences later.

**Thank you for your careful observation. We appreciate your suggestion to remove the part of the sentence in lines 13-15 that implies uncertainty about the relation between resistance and the effectiveness of SMC. Upon further analysis, however, we feel that there is no contradiction because chemoprevention is different from treatment. Indeed, SP is no longer recommended for the treatment of malaria episodes, but it’s still recommended for chemoprevention in pregnancy despite high resistance. That is what we mean by the “relation between resistance and effectiveness of SMC.”**

 Recruitment and data collection: Line 6: is it only one child or one child per household?

**Thank you for seeking clarification on this point. We meant one eligible child from each household as stated.**

 Line 10: what were the end of round demographic survey results used for?

**Thank you for your question regarding the use of the end of round demographic survey results mentioned in line 10. By incorporating the end of round demographic survey, we aimed to provide information on coverage and quality, suitability of design, the extent of attainment of targets, and assess planning and general implementation of processes as required in the SMC protocol**

 Process evaluation component: Line 5 typo: possible60; line 9: "progresses" not "progressed".

**Thank you for catching those typos in the process evaluation component. We appreciate your attention to detail. We will make the necessary corrections in line 5, changing "possible" to "possible, ," and in line 9, replacing "progressed" with "progresses."**

 This paper would be stronger and more complete if the final data results would be available. If this is not ready, I'm not clear if the objective of the study has been achieved or if results to substantiate the objectives can be proven.

**Thank you for your feedback. We understand your concern about the completeness and strength of the paper without access to the final data results. Please note that this is a protocol paper that was submitted at the time when data collection was not complete.**

**While the article currently lacks the final data results, we want to assure you that the study's objectives have been pursued rigorously and with meticulous methodology. The data collection processes have since been completed, and we aim to present comprehensive and conclusive results in future publications.**

**We acknowledge the importance of substantiating the study's objectives with concrete evidence, and we will work diligently to finalize the data analysis and interpretation. Once the results are available, we will consider publishing a follow-up paper to share the outcomes and their implications.**

**Thank you for your understanding and valuable input.**
